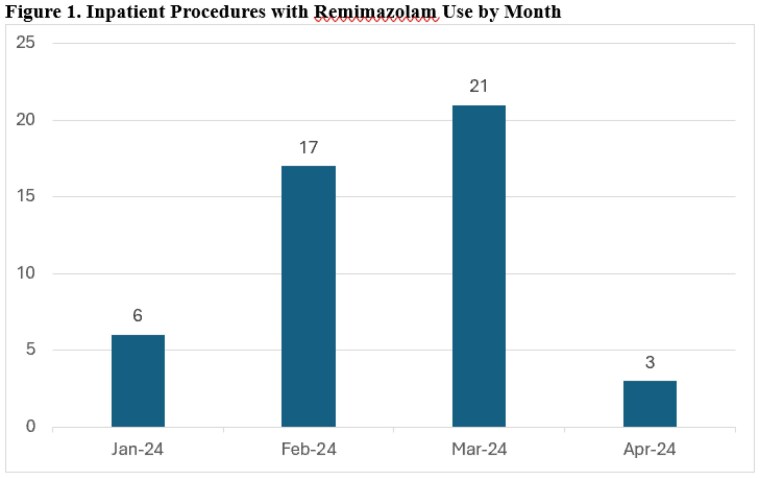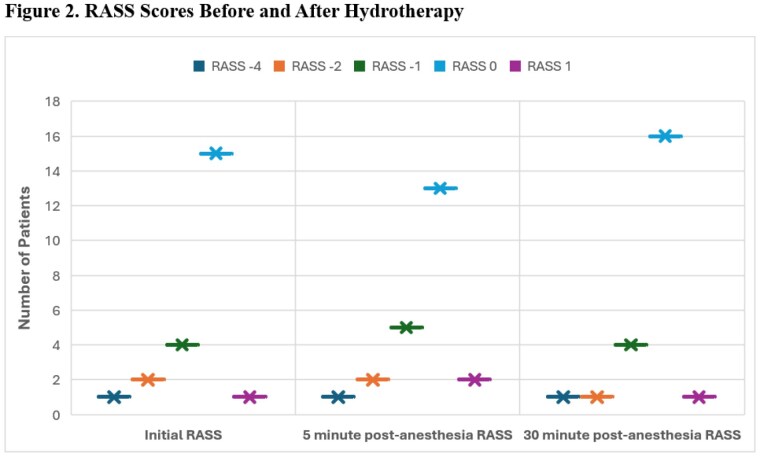# 49 Use of Remimazolam Besylate for Procedural Sedation During Dressing Changes in the Burn ICU

**DOI:** 10.1093/jbcr/iraf019.049

**Published:** 2025-04-01

**Authors:** Michael Martz, Rishi Patel, Steven Kahn

**Affiliations:** South Carolina Burn Center, Medical University of South Carolina; Medical University of South Carolina; Medical University of South Carolina

## Abstract

**Introduction:**

Acute procedural pain and tolerance of burn wound care remains a major challenge for patients and healthcare professionals alike. Anesthesia professionals are effective at providing safe sedation during dressing changes; however, daily dressings changes with anesthesia can lead to unwanted effects such as prolonged post-procedural sedation and increased levels of delirium. Remimazolam is a novel ultrashort-acting benzodiazepine that was approved by the FDA in July 2020, which produces sedation by acting as a modulator of the gamma-amino butyric acid-A (GABA-A) receptor. The purpose of this study was to determine feasibility, safety, and the time of offset when remimazolam was used for burn procedural sedation.

**Methods:**

This was a single center analysis of prospectively collected data using remimazolam for procedural sedation during “hydrotherapy” burn wound care sessions in the burn intensive care unit. Data was collected during a trial period of the drug between January 2024 and May 2024. In addition, patient demographics, remimazolam doses, duration of administration, concomitant anesthetic agents were collected. Richmond Agitation Sedation Scale (RASS) scores were documented prior to anesthesia, 5 minutes post-anesthesia stop, and 30 minutes post-anesthesia. Data was reported with descriptive statistics.

**Results:**

During the study period, remimazolam was utilized 47 times across 8 unique patients (Figure 1). The median dose of remimazolam per sedation session was 20 mg (IQR 20 – 27.5 mg). Doses ranged from 2.5mg to 40 mg. Thirteen cases used a total dose greater than 20mg. Other sedative medications were used in 29 cases (62%), including dexmedetomidine (14 cases, 30%), ketamine (20 cases, 42%), and propofol (9 cases, 19%). While precise procedural duration was not documented for all 44 cases, the mean time from first to last dose of remimazolam was 30 minutes (standard deviation [SD] 18).

Additional data was tracked for 15 of these hydrotherapy cases where remimazolam was used for sedation. The mean duration of these 15 cases was 65.2 minutes (SD 22). In two cases, it was noted that dexmedetomidine and/or propofol were used due to longer than expected procedures. The median RASS score was 0 at all 3 time points. All 14 patients (100%) with available post-anesthesia scores returned to their baseline RASS score 5 minutes post-anesthesia stop (Figure 2).

**Conclusions:**

This study aimed at evaluating a single center’s experience with utilizing a novel drug, Remimazolam, during procedural sedation for burn wound care. In addition to prompt recovery of RASS scores, providers empirically noted rapid reversal of sedation after wound care sessions.

**Applicability of Research to Practice:**

Given the unique properties of the drug, potential benefits may include rapid resolution of sedation, decreased delirium, increased post-sedation participation in therapies, and minimal interruptions in nutrition after sedation- which all warrant further study.

**Funding for the Study:**

N/A